# Analysis of oceanic suspended particulate matter in the western North Pacific using the complex amplitude sensor

**DOI:** 10.1038/s41598-024-70683-1

**Published:** 2024-08-29

**Authors:** Atsushi Yoshida, Yutaka Tobo, Kouji Adachi, Nobuhiro Moteki, Yoshimi Kawai, Kosei Sasaoka, Makoto Koike

**Affiliations:** 1 National Institute of Polar Research, Tachikawa, Tokyo Japan; 2 Graduate Institute for Advanced Studies, SOKENDAI, Tachikawa, Tokyo Japan; 3 Meteorological Research Institute, Tsukuba, Ibaraki Japan; 4 Tokyo Metropolitan University, Hachioji, Tokyo Japan; 5 Japan Agency for Marine-Earth Science and Technology, Yokosuka, Kanagawa Japan; 6 The University of Tokyo, Tokyo, Japan

**Keywords:** Element cycles, Marine chemistry, Atmospheric chemistry

## Abstract

Oceanic suspended particulate matter (SPM) plays important roles in the coupling of climate and biogeochemical cycles via ocean–atmosphere interactions. However, methods for quantifying the properties of SPM in seawater have not yet been well established. Here we present the application of the recently developed complex amplitude sensor (CAS) for analyzing the complex forward-scattering amplitude of individual SPM (0.2–5.0 µm in diameter) obtained at depths of 0–100 m during a research cruise in the western North Pacific. The measured distribution of the complex amplitude indicated that the CAS-derived SPM data could be roughly classified into five major types. Comparison with reference sample’s complex amplitude data and scanning electron microscopy analysis suggested that these types could be attributed mainly to diatom fragments, carbonaceous materials (likely organic matter), mineral dusts, iron oxides, or black carbon. Depth profiles revealed that relatively high concentrations of SPM, presumably dominated by diatom fragments and carbonaceous materials with peak diameters of 0.7–1.0 µm, were typically associated with elevated turbidities and chlorophyll *a* concentrations. Based on this case study, we discuss the practical advantages and limitations of using the CAS to measure size-resolved concentrations of SPM in seawater and to characterize its composition.

## Introduction

Suspended particulate matter (SPM) is ubiquitous in the ocean and plays a significant role in the biogeochemical cycling of elements in marine environments. The sources of SPM in the ocean can be categorized as either biogenic generated through marine biological activity (e.g., production of SPM associated with phytoplankton, such as diatoms, coccolithophores, dinoflagellates, and cyanobacteria) or external, such as aerosols deposited from the atmosphere (e.g., aerosols released from terrestrial sources) and input from river runoff^1,2^. Following the biogenic formation or external input, SPM in the ocean undergoes subsequent decomposition or sinking. The decomposition of SPM is an important source of nutrients in the surface ocean, while the sinking of SPM into the ocean interior is a crucial process for carbon removal from the atmosphere^2^. The direct emission of marine SPM into the atmosphere is thought to be an important source of atmospheric aerosols^3^. Such marine aerosols can act as cloud condensation nuclei, and some of them may also serve as efficient ice nucleating particles^4^. Thus, SPM plays important roles in the coupling of climate and biogeochemical cycles via ocean–atmosphere interactions.

Measurements of SPM concentrations and characteristics are an important part of investigations of its roles in climate and biogeochemical cycles. Measuring turbidity is a common method to monitor SPM abundance^5,6^. Turbidity is quantified based on the amount of light scattered by particles in water, and turbidity is thus a proxy of the bulk mass of SPM^7^. In marine observations, vertical profiles of turbidity in the water column are often measured using optical backscatter sensors mounted on a conductivity–temperature–depth (CTD) system^8^. Another indicator of the amount of SPM is the concentration of chlorophyll *a* (Chl-*a*), which is a good proxy of the abundance of phytoplankton since phytoplankton species contain plant pigments and Chl-*a* is the primary photosynthetic pigment present in all forms of algae. Increase of the concentration of Chl-*a* in surface water is often attributed to bloom-forming phytoplankton such as diatoms and coccolithophores^9,10^. Measurements of the concentrations of specific elements or substances are also commonly used to characterize SPM. For example, particulate organic carbon, nitrogen, and phosphorus collected on a filter are oxidized and their concentrations measured with an infrared anlyzer^11^ or a colorimetric technique^12^. The concentrations of gel-like organic particles such as transparent exopolymer particles can be estimated using staining and light absorption measurements^13^. The above methods provide a bulk analysis of SPM and do not provide information about the concentration of SPM as a function of particle size.

Information about the particle size-resolved concentrations of SPM is important because particle size is essential for determining the sinking rates of particles in the ocean and atmosphere^3,14^, and it thus affects vertical and horizontal fluxes. Particles size is also an important determinant of ice nucleating ability^15^. Techniques that measure individual particles suspended in water have been used to estimate size-resolved concentrations of particles^16^. For example, the Coulter Counter, which is an electrical instrument that counts particles, is widely used in marine science^17,18^. Optical methods, which can be used to measure the signal of scattered light from individual particles illuminated by a light source, are also widely used for particle-sizing. The flow cytometer uses a laser beam to illuminate individual particles in a suspension of particles and measures the scattered and fluorescent light to discriminate phytoplankton^19–21^. However, these methods may not be suitable for quantifying both the size-resolved concentrations and specific types of SPM in seawater, particularly non-biological particles.

In this study, we evaluated the applicability of a complex amplitude sensor (CAS^22^) for particle-sizing and characterization of SPM. The CAS can measure the complex forward-scattering amplitude (complex amplitude *S*) of single particles suspended in a fluid flow. Yoshida et al*.*^23^ have demonstrated that the measured value of *S* for mineral dust differ from that of black carbon (BC), iron oxides, and bacteria. They have further shown that the size-resolved concentrations of mineral dust can be estimated from *S* data. Ohata et al*.*^24^ have measured the *S* of atmospheric water-insoluble aerosols; characterized them as either BC-like, mineral-dust-like, or primary biological particles; and quantified the concentration of each kind of aerosol. Moteki et al*.*^25^ have identified ambient BC aerosols and have constrained their complex refractive index from *S* data. Based on these studies, we hypothesized that *S* measurements could also be used to measure the size-resolved concentrations and composition of SPM in seawater.

In this study, we evaluated the capability of CAS to identify particle composition and quantify size-resolved concentrations of oceanic SPM. For this purpose, we characterized the *S* data of SPM in seawater and compared them with those of reference materials with known compositions. We also used data from electron microscopic analyses of sampled SPMs to evaluate their composition. Based on these studies, we also show the size-resolved concentrations and depth profiles of SPM. Finally, we discuss the potentials and challenges of using a CAS to measure the concentrations and characteristics of SPM in seawater.

## Methods

### SPM samples

**Fig. 1:**
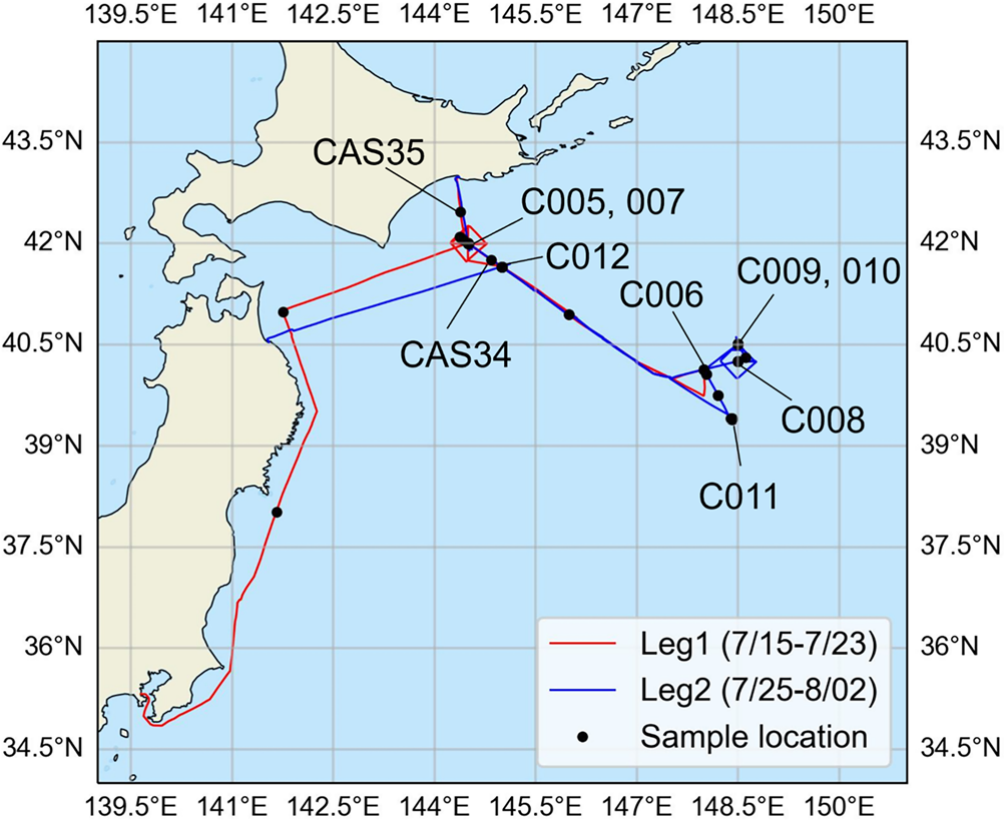
Research area and track of cruise. The locations of collecting seawater samples are denoted as black points. This figure was generated by Matplotlib (version 3.8.0, Hunter, J. D., Matplotlib: a 2D graphics environment. Computing in Science and Engineering 9, 2007, https://ieeexplore.ieee.org/document/4160265) and Cartopy (version 0.21.1, MetOffice, UK, https://scitools.org.uk/cartopy/docs/latest/index.html).

Seawater samples were collected on a research cruise on board R/V *Shinsei-maru* from 15 July to 2 August 2022 in southeast off Hokkaido, the western North Pacific (Cruise ID: KS-22-10). The seawater in this area is influenced by both the Oyashio Current, a nutrient-rich and cold subarctic ocean current characterized by high biological production, and the Kuroshio Current, a warm ocean current characterized by relatively low biological production. Intensive CTD observations and seawater sampling were conducted at stations C005–C012 (Fig. 1). The temperature–salinity (T–S) diagrams at C005 and C011 were close to that of the Oyashio Current, whereas those at C006, 007, 008, 009, 010, and 012 were close to that of Kuroshio Current (not shown).

Seawater was collected in 2000-mL dark bottles in the following three ways: bucket sampling at the surface (11 samples); a CTD sampling system with Niskin bottles at depths of 10 (9.8–10.5), 20 (18.7–22.8), 30 (29.6–37.7), 50 (48.9–56.1), and 100 (99.1–100.0) m (40 samples); and underway sampling from an intake at a depth of ~ 3 m (25 samples). At station C005, CAS measurements were conducted only for samples collected from depths of 37.7 m or less. Underway sampling was conducted in addition to CTD observations when the vessel was moving. The dark bottles were rinsed twice with seawater prior to sampling. After sampling, we removed large particles with a 12-µm pore size Whatman Nuclepore track-etched membrane filter (47-mm diameter) because such large particles would clog the flow cells (thickness of flow pass = 50 µm) in the CAS instrument. We conducted two types of CAS measurements. One type was “in situ” measurements that were conducted on board immediately (within 4 h) after collecting seawater. Another was “filter” measurements. To perform filter measurements, we first removed large particles from the seawater with the 12-µm pore size filter and concentrated the SPM in the seawater (0.5–1.5 L) on a 0.4-µm pore size Nuclepore track-etched membrane filter (47-mm diameter) and then dried them on a clean bench. The dried samples were transferred to 10-mL sterile centrifuge tubes and stored at ~ 4 °C in the laboratory. These filter samples were immersed in 25 mL of laboratory-purified water (AS ONE Corp.) in a glass vial for measurements with the CAS instrument. We sonicated at frequencies of 28, 45, and 120 kHz for 20 s each (1 min total) to suspend the particles. Finally, the suspension was introduced into the CAS instrument. We also prepared additional filter samples, CAS34 and CAS35, to compare the results of filter measurements made with the CAS, a scanning electron microscope (SEM) coupled with energy-dispersive X-ray spectrometer (EDS), and Coulter Counter. The CAS34 and CAS35 samples were prepared from seawater collected via underway sampling (Fig. 1).

We monitored turbidity with a Seapoint Turbidity Meter (Seapoint Sensors, Inc.) mounted on the CTD frame. Seawater samples for Chl-*a* analysis were collected from Niskin bottles or (at the surface) a bucket and transferred to 250-mL brown polyethylene bottles. Chl-*a* samples were gently filtered (volume = 200 mL) by low vacuum pressure (< 0.02 MPa) through a Whatman GF/F filter (25-mm diameter) in a dark room. The filter samples were stored at − 20 °C in the dark. The Chl-*a* was extracted in 7 mL of *N*,*N*-dimethylformamide (DMF) for more than 24 h. After extraction, Chl-*a* concentrations were measured with a Turner fluorometer (10-AU, Turner Designs), which had previously been calibrated against a pure Chl-*a* standard (Sigma-Aldrich Co., LLC). We used the fluorometric non-acidification method^26^ to estimate Chl-*a* concentrations. Vertical profiles of turbidity and Chl-*a* concentrations were compared with those of SPM concentrations measured by the CAS instrument.

### References samples

We prepared some reference samples for comparison of *S* data. Specifically, we used reference mineral dust samples including NX illite (illite-rich mineral dust^27^), quartz (SiO_2_-rich mineral dust, JIS Z 8901 Class3, Association of Powder Process Industry and Engineering), and calcium carbonate (CaCO_3_, Cat. No. CAH28PB, Kojundo Chemical Laboratory Co., Ltd.). We used iron oxide samples including hematite (Fe_2_O_3_, Lot No. 180611, Toda Kogyo Corp.) and goethite (FeOOH, Lot No. 180611, Toda Kogyo Corp.). We used Snomax powder, which is made from the bacterium *Pseudomonas syringae*^28^, as a reference for bacteria. We used NIES4227, a strain of *Thalassiosira nordenskioeldii Cleve* provided by the National Institute for Environmental Studies (NIES) collection through the National BioResource Project (NBRP) of the Ministry of Education, Culture, Sports, Science and Technology, Japan, to obtain reference data for a diatom. This strain was isolated from the Oyashio Current in the western North Pacific^29^. It is possible that this culture contained certain amounts of contaminants (e.g., culture medium) other than the cultured strain of *Thalassiosira*. We also obtained *S* data for ambient BC aerosols that we collected onboard the same research cruise and measured with the CAS instrument^25^ for identifying BC particles deposited from the atmosphere to the sea surface.

### CAS instrument

The CAS measures the complex forward-scattering amplitude of single particles suspended in a fluid flow at a wavelength of 632.8 nm. The forward complex amplitude $S\left(\theta =0^\circ \right)=\text{Re}S+\text{Im}S=\left|S\right|{e}^{i\Delta }$ represents the amplitude and phase shift of the scattered field observed at a forward angle relative to the incident plane-wave field. The *S* parameter reflects the physical properties of particles such as their complex refractive index, volume, shape, and orientation^22,30^.

The optical system of the CAS used in this study was identical to that used by Moteki et al.^25^ The CAS consists of two channels, namely the s-channel for measuring sub-micron particles (0.2–1.0 µm) and the l-channel for super-micron particles (1.0–5.0 µm). Each channel consists of optical components (e.g., mirrors, lens, and beam expander) and a flow cell. Particles suspended in freshwater or seawater are introduced into the flow cell using a peristaltic pump. The flow rate is monitored by a flow meter (SLI 2000, Sensirion) and maintained at ~ 1.9 mL/min. Individual particles are detected when they pass near the focused area of the laser beam (λ = 632.8 nm). The interference of the light scattered forward by individual particles with the incident light (laser beam) is detected by a four-segment photodiode. By analyzing the four interference signals, the complex amplitude *S* of individual particles is determined. To estimate the size of the beam waist spot size, which needs to be calibrated to determine the *S* value from the signals, we used the method described by Moteki^22^ and measured polystyrene latex standard particles with sizes of 0.303, 0.510, 0.803, 1.036, 2.020, 2.994, and 5.010 µm (PSL, Thermo Scientific, 3000 and 4000 series). The resulting beam waist values for the s- and l-channels were estimated to be 3.13 µm and 13.9 µm, respectively. Before each CAS measurement, we introduced Milli-Q water or pure water into the CAS instrument to clean the flow tubes and flow cells. The sample measurement was initiated after the particle detection rate had decreased to less than 0.1 s^−1^.

### Determination of number concentrations

The number concentration of SPM detected by the CAS instrument in fluid water (*C* [m^−3^]) is expressed by the following equation^24^:

1 $$C=\frac{R}{AU} ,$$

where *R* (s^−1^) is the particle detection rate after correction by the duty cycle of the computer of the CAS instrument for recording particle signal data, *A* (m^2^) is the effective detection area perpendicular to the sample flow in the CAS flow cell, and *U* (ms^−1^) is the average velocity of the sample water in the flow cell. The effective detection area *A* depends on the alignment of optical systems and thresholds set in the software to detect waveform signals of particles passing near the center of the laser beam. The thresholds were identical to those set by Moteki^25^. We experimentally determined the value of *A* by measuring *R* for suspensions of various sizes of PSLs. The number concentrations of which, *C*, were determined based on the Lambert–Beer law^31^. We used PSLs with sizes of 0.303, 0.510, 0.803, 1.036, 2.020, 2.994, and 5.010 µm. Supplementary Fig. 1 shows the experimentally determined values of *A* in the s-channel (*A*_s-ch_) and the l-channel (*A*_l-ch_). The *A*_s-ch_ for 0.303-µm PSLs was 2.82 × 10^−11^ m^2^, and the mean value for 0.510-, 0.803-, and 1.036-µm PSLs was 4.52 × 10^−11^ m^2^. The mean value of *A*_l-ch_ for 0.803- and 1.036-µm PSLs was 1.50 × 10^−10^ m^2^, and that of 2.020-, 2.994-, and 5.010-µm PSLs was 2.38 × 10^−10^ m^2^. In both channels, the *A* values were low for the smaller PSL. A low signal-to-noise (S/N) ratio of waveform signals for such small particles could lead to failure to detect the particle signal and decrease the *A* value^24^. The size dependence of *A* is likely to vary as a function of particle composition because the relationship between the S/N ratio of the signal and particle size varies among particle compositions. For this reason, we used *A*_s-ch_ = 4.52 × 10^−11^ m^2^ and *A*_l-ch_ = 2.38 × 10^−10^ m^2^ and did not consider the lower *A* value determined by the smaller particles when measuring the SPM. The derived number concentration for small SPMs (< ~ 0.5 µm for s-channel and < ~ 1.0 µm for l-channel) was thus likely underestimated.

To validate the procedure for determining the number concentration, we conducted simultaneous measurements of PSL suspensions by CAS and Coulter Counter (Multisizer 4, Beckman Coulter, Inc.) in the laboratory. The aperture size of the Coulter Counter was 30 µm, and the detectable particle size range was 0.6–24 µm. We prepared monodispersed suspensions containing 1.036-µm PSLs and polydispersed suspensions containing 0.803-, 1.036-, and 2.994-µm PSLs. We diluted these suspensions with ISOTON II electrolyte to measure electrical impedance with a Coulter Counter. Because the PSL particles were spherical and their complex refractive index *m* had been measured, it was straightforward to determine their particle size from measured *S* data points. We first calculated the theoretical *S* curve, which is a series of points of (Re*S*, Im*S*) with diameter *D* as parameter, based on Mie theory, assuming the complex refractive index of a PSL in vacuum at λ = 632.8 nm to be 1.5854 + 6.1764 × 10^−7^i based on experimental data^32^. We then projected each measured data point on the *S* curve to determine the particle size. Combining the particle size and particle detection rate allowed us to determine the size-resolved concentration. Supplementary Fig. 2a,b show size-resolved concentrations, d*N*/dlog*D*, where *D* represents the diameter of the particle and d*N* represents the number concentration of particles within each size bin (dlog*D*). Both the CAS and Coulter Counter successfully quantified the sizes of the PSLs. We further derived the number concentration *N* for PSLs of *D* = 0.803, 1.036, and 2.994 µm by integrating d*N*/dlog*D* in the *D* ranges of 0.6–0.86, 0.86–1.2, and 2.6–3.5 µm, respectively. Supplementary Fig. 2c shows the scatter plot of the *N* derived by the CAS and Coulter Counter. For the l-channel, we also derived a modified *N* for PSLs of *D* = 0.803 and 1.036 µm by using an *A*_l-ch_ value of 1.50 × 10^−10^ m^2^ instead of 2.38 × 10^−10^ m^2^. Use of the modified *N* caused the data points to lie close to the 1:1 line in the scatter plot. Combining the data points for the s-channel and the modified l-channel led to a linear-fit slope of 0.8727 and an extremely high *R*^2^ value of 0.996. We obtained good agreement between the size-resolved concentrations of PSL determined by CAS and Coulter Counter.

### Characterization of particle composition and quantification of particle size for SPM

The complex amplitude of *S* reflects the physical properties of particles, including their complex refractive index, size, and shape. It is expected that a group of particles with a specific composition will form a characteristic *S* cluster on the complex *S* plane^23–25,30,33^. Because there is some arbitrariness in how observed data are divided into clusters, we set boundaries on the complex *S* plane to classify the clusters for the SPM. To determine the particle size for each *S* data point, we used a forward model that calculates the *S* value from the assumed complex refractive index and shape. Moteki et al.^25^ estimated the complex refractive index of ambient BC aerosols measured by CAS onboard the same research cruise. They used a curve fit to the *S* data and then conducted Bayesian inference with the SPHPACK shape model to determine the most plausible parameter vector (size, complex refractive index, and shape) for the *S* data points on the fitted curve. We used the relation between the *S* value and its plausible particle size expressed as the volume equivalent diameter inferred by Moteki et al.^25^ based on the data measured on 27 July 2022 (Fig. 7a from Moteki et al.^25^) to determine the particle size of the BC in the SPM in seawater. On the other hand, detailed forward models that fully consider the particle’s physical properties (volume, shape, and complex refractive index) have not yet been developed for non-BC SPMs. We estimated the size of non-BC particles by assuming a spherical shape and zero imaginary part of the complex refractive index (i.e., a non-absorbing sphere). In this approach, we prepared a table of theoretical *S* values for non-absorbing spheres with diameters in the range 0.1 µm < *D* < 10.0 µm and refractive indices in the range 1.34 < *m* < 2.40 using the Mie theory, where *m* is the complex refractive index of particles in vacuum. We then determined the most likely refractive index *m*_ml_ and diameter *D* of each measured particle by minimizing the distance between the measured and theoretical *S* values. We note that *S* depends on the ratio of the complex refractive index of particles in vacuum *m* to that of the medium of the suspension *m*_*med*_. In calculating the theoretical *S* values, we assumed *m*_*med*_ to be the refractive index of pure water for the filter measurements and seawater for the in situ measurements. We used *m*_*med*_ = 1.33154 for pure water at λ = 632.8 nm^34^ and *m*_*med*_ = 1.3373 for seawater at λ = 640 nm, temperature = 20 °C, and salinity of 34.0‰^35^. For each cluster, a whole size-resolved number concentration was constructed by combining the results from the s- and l-channels. The detectable ranges of *S* partly overlapped between the s- and l-channel configurations. In this study, data points detected in the s-channel were used in the range 0 µm < Re*S* < 1.5 µm and 0 µm < Im*S* < 0.5 µm, and those detected in the l-channel were used in the range 1.5 µm < Re*S* < 10.0 µm and 0.5 µm < Im*S* < 5.0 µm. The reason for setting the upper limits for the l-channel was that large particles that exceeded the upper limit did not satisfy the validity criteria of the plane wave approximation when the *S* value was determined from the signal waveform^22^. All computational analyses of *S* data for characterizing particle composition and quantifying size-resolved concentration were carried out using the Python programming language (version 3.10.12, https://www.python.org).

To identify the particle compositions corresponding to the *S* clusters, we analyzed CAS34 and CAS35 samples using a SEM (SU 3500, Hitachi High-Technologies Co.) coupled with an EDS (X-Max50, Horiba, Ltd.). Individual particles over the filters having contrast above the threshold intensity in the SEM images were automatically detected, and their area equivalent diameter (> 0.55 µm) and elemental spectra were measured using multipoint analysis software (Oxford INCA software). Based on the elemental spectra, we classified particles into specific types and determined each size distribution.

## Results and discussion

### Particle composition obtained by CAS measurement

#### Complex amplitude data

**Fig. 2:**
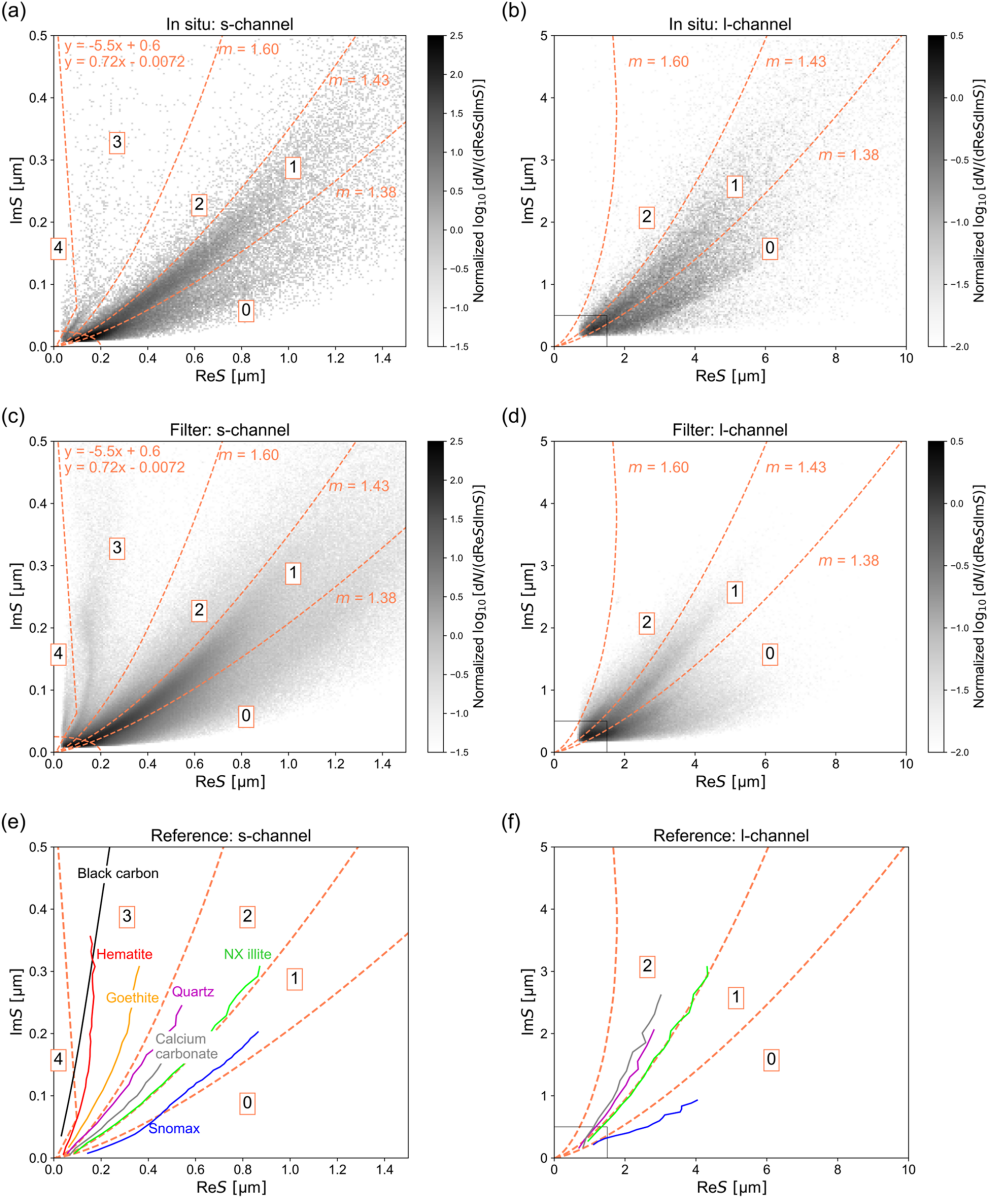
(**a–d**) Distribution of *S* data points combined for (**a**,**b**) all in situ samples and (**c**,**d**) all filter samples. Boundaries for classifying *S* data points are represented as dashed curves and lines. The boundary curves are theoretical *S* curves for sphere particles with complex refractive index of *m*. (**e**,**f**) Median of Re*S* values as functions of Im*S* values for reference samples. The black rectangle boxes at lower left in l-channel panels show the outer boundaries of s-channel panels.

Figure 2a–d shows the *S* data points for both in situ and filter measurements with all SPM samples collected from this study. The distribution of *S* data points was expressed as normalized d*N*/(dRe*S*dIm*S*), where d*N* (mL^−1^) was the number of particles within each grid (dRe*S*dIm*S*) on the complex *S* plane. When characterizing the data points for the s-channel, we excluded the data points in the boundary defined by the elliptic function (x/0.2)^2^ + (y/0.025)^2^ = 1, where x = Re*S* and y = Im*S*, because the data points near the origin of the complex plane were too concentrated to classify particle Types. We categorized the other data points into Types 0–4 as follows. We classified the data points located to the right of the theoretical *S* curve for the sphere with *m* = 1.38, between *m* = 1.38 and 1.43, and between *m* = 1.43 and 1.60 as Types 0, 1, and 2, respectively, as denoted in Fig. 2. These theoretical curves were calculated based on Mie theory. In the s-channel, we further confirmed some data points distributed to the left of *m* = 1.60. We classified those data points as Type 3 if they were located to the right of the lines y = − 5.5x + 0.6 or y = 0.72x − 0.0072, where x = Re*S* and y = Im*S*. We also classified the data points as Type 4 if they were located to the left of both of these lines. We did not define Types 3 and 4 for the l-channel because of the limited number of data points. Among all samples measured by in situ methods, the 25th–75th percentiles of the number concentrations of Types 0, 1, 2, 3, and 4 data points combined in both the s- and l-channels were 2.4 × 10^4^–6.7 × 10^4^, 4.8 × 10^4^–9.7 × 10^4^, 6.0 × 10^3^–1.5 × 10^4^, 3.8 × 10^2^–1.2 × 10^3^, 1.5 × 10^2^–4.1 × 10^2^ mL^−1^, respectively. For the filter measurements, the corresponding values were 1.3 × 10^4^–3.7 × 10^4^, 1.5 × 10^4^–4.8 × 10^4^, 4.7 × 10^3^–1.4 × 10^4^, 9.3 × 10^2^–5.3 × 10^3^, 2.7 × 10^1^–1.8 × 10^2^ mL^−1^, respectively.

Figure 2e,f present the *S* for the reference samples, which are depicted as median Re*S* values as functions of Im*S*. The *S* data points for Snomax were concentrated near the boundary between Types 0 and 1 (i.e., around *m* = 1.38) in the s-channel, and they were predominantly distributed in the Type 0 region in the l-channel. The data points for NX illite were distributed near the boundary between Types 1 and 2 in both the s- and l-channels (i.e., around *m* = 1.43). Most data points for the calcium carbonate and quartz samples were distributed in Type 2 regions in both the s- and l- channels. Most data points of the hematite and goethite were distributed in the Type 3 region in the s-channel. Most data points for small BC particles (i.e., Im*S* < 0.15 µm) were distributed in the Type 4 region, whereas the data for larger BC particles (i.e., Im*S* > 0.15 µm) were distributed in the Type 3 region. The distinct distributions of the smaller BC and iron oxide made it necessary to separate Types 3 and 4. Although the data points for diatom culture strain NIES4227 exhibited a scattered distribution (Supplementary Fig. 3), 56% of the data points were classified as Type 0, whereas 19%, 19%, 4.6%, and 0.25% of the data points were classified as Types 1, 2, 3, 4, respectively, when the data from the s- and l-channels were combined.

#### CAS34 and CAS35 samples

**Fig. 3:**
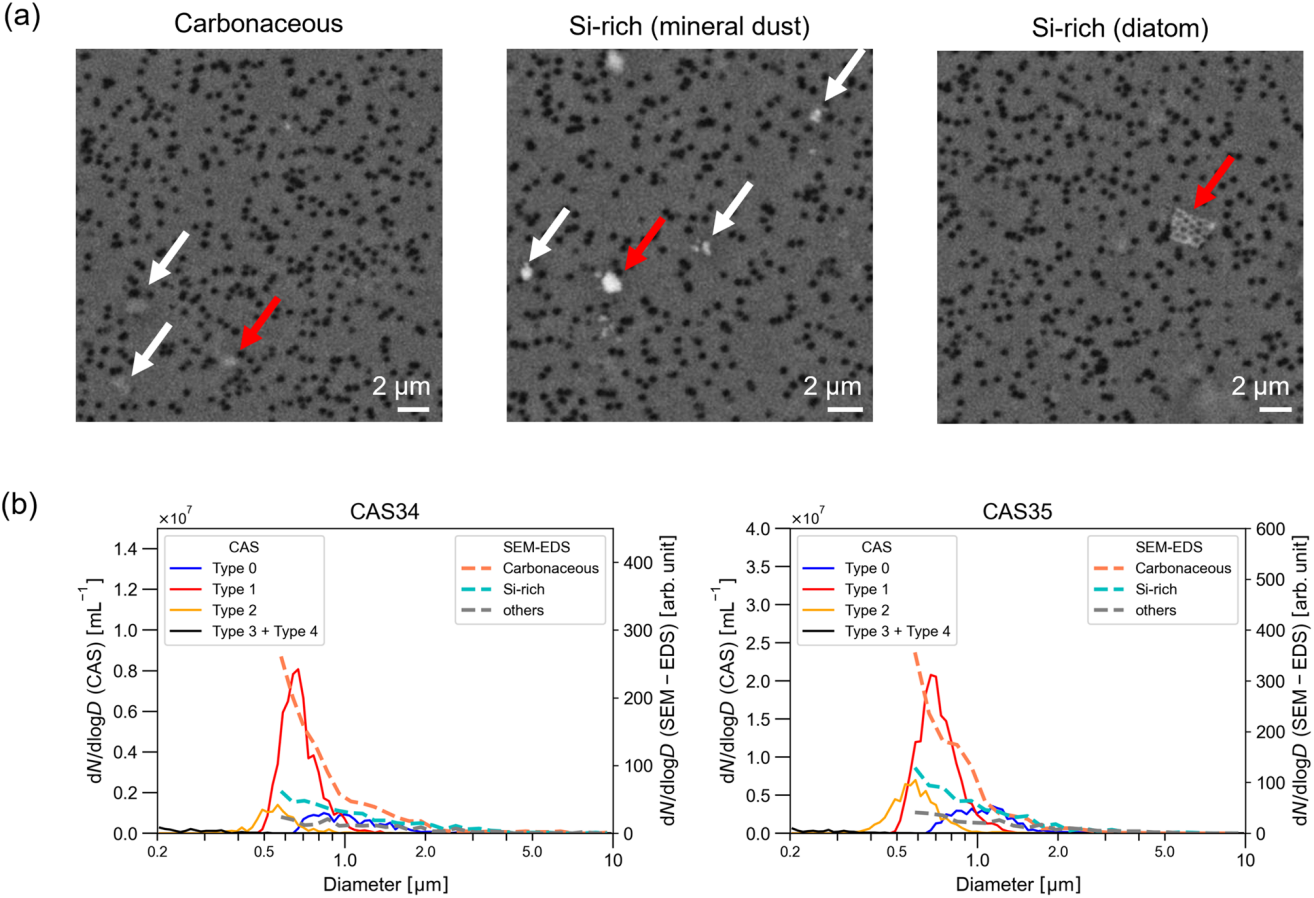
SEM–EDS analyses of representative particles found in CAS34 and CAS35 samples. (**a**) Representative SEM images of carbonaceous and Si-rich (mineral dust and diatom) particles (indicated by arrows). Red arrows indicate particles whose spectra are shown in Supplementary Fig. 4. (**b**) Comparison of size-resolved concentrations of the particles determined by CAS and SEM–EDS.

Figure 3a presents representative SEM images of the analyzed particles for CAS34 and CAS35. Representative EDS spectra are shown in Supplementary Fig. 4. Because the EDS spectra contained background signals attributable to carbon (C) and oxygen (O) from the polycarbonate filter and aluminum (Al) from the stage holder, it was difficult to accurately quantify the signals of C, O, and Al from the particles. Based on these spectra, we categorized the detected particles into several types. We classified more than half of the particles (64% for CAS34 and 57% for CAS35) as “carbonaceous.” Weak signals from nitrogen (N) tended to be commonly present in the carbonaceous particles (Supplementary Fig. 4) and signals from phosphorous (P) were also detected in some of them. Because phosphorus and nitrogen are elements that make up biological organic compounds^36^, we hypothesized that these carbonaceous particles were mainly attributable to biological organic compounds. We classified particles from which there were strong signals of silicon (Si) as “Si-rich” particles, and they accounted for 26% (CAS34) and 29% (CAS35) of the total analyzed particles. SEM images of Si-rich particles (Fig. 3a) indicated that they might be composed of mineral dust or diatoms. Mineral dust particles typically exhibit angular shapes with a brighter appearance in back-scattered electron images because of their high density. Some Si-rich particles in the super-micron size range had shapes characteristic of diatoms (Fig. 3a right). However, we did not distinguish between mineral dust and diatoms because it was difficult for our SEM–EDS analysis to unambiguously distinguish between small (< ~ 1 µm) silicate mineral dust and a fragment of diatom based on their shapes and compositions. Particles from which we detected strong signals from other elements such as sodium, potassium, and chlorine were classified as “Others” and accounted for 11% (CAS34) and 13% (CAS35) of all analyzed particles.

We compared the size-resolved number concentration of each Type derived from CAS based on the boundaries defined above and SEM–EDS (Fig. 3b). We determined the CAS-derived sizes of Type 4 particles by assuming that they were BC (see the next section) and used the relationship between the *S* value and volume-equivalent diameter inferred by Moteki et al.^25^ For the sizes of Types 0–3, we assumed that these particles were non-absorbing spheres. We acknowledge a significant uncertainty in estimating the particle size of Type 3 particles (likely light-absorbing iron oxides, see the next section) on the assumption that they are non-absorbing spheres, and thus more accurate estimation of the particle size would require a suitable model that simulates the *S* for realistic iron oxide particles. Nevertheless, the combined size-resolved concentrations of Types 3 and 4 (Type 3 + Type 4) were so low that they were difficult to discern in the plots. We compared the CAS results with the size-resolved concentrations of carbonaceous and Si-rich types determined by SEM–EDS analysis. Note that the size-resolved concentrations derived from the SEM–EDS analysis provide only the relative concentrations of SPM in the seawater. For both CAS34 and CAS35 samples in the size range 0.55–5.0 µm, the Type 1 (carbonaceous) particles were approximately 2.5-fold and 1.9-fold more abundant than the Type 0 + Type 2 (Si-rich) particles, respectively. The size distributions of the Type 1 (carbonaceous) particles exhibited a steep decrease from 0.55 to 1.0 µm, whereas the Type 0 + Type 2 (Si-rich) particles exhibited a gradual decrease. These distributions of Type 0 + Type 2 and Type 1 were similar to those of Si-rich particles and carbonaceous particles measured using SEM, respectively.

In Supplementary Fig. 5, we compare the absolute number concentrations for CAS34 samples determined by CAS and Coulter Counter. Despite the differences of the measuring principles between the CAS and Coulter Counter, the size-resolved concentrations derived from both instruments were consistent for particles larger than 0.6 µm.

#### Composition of particle types for SPMs obtained by CAS

Based on the above results, we propose the Major, Minor, and Possible compositions corresponding to each Type (Table 1). For all Types, the Major composition of the adjacent Type on the complex *S* plane are included in the Minor composition. A composition, which may be either the Major or Minor composition, but there is no clear evidence from this study, is categorized as the Possible composition.

**Table 1:** Particle composition of Types 0–4 proposed in this study.

Type	Major	Minor	Possible
Type 0	Diatom fragments Carbonaceous		
Type 1	Carbonaceous	Diatom fragments, mineral dust	
Type 2	Mineral dust	Carbonaceous, diatom fragments, iron oxides	Coccolith
Type 3	Iron oxides	Mineral dust, black carbon	
Type 4	Black carbon	Iron oxides	

There are several candidates for the Major composition of Type 0. For example, bacteria are carbonaceous, and the *S* data points of Snomax were distributed in the regions of both Type 0 and 1. For this reason, we included carbonaceous particles as a Major composition of Type 0 as well as Type 1. In addition, we assumed diatom fragments to be a Major composition of Type 0 because more than 50% of the *S* data points of the NIES4227 sample were classified as Type 0 (Supplementary Fig. 3). In contrast, ~ 20% of the *S* data points for NIES4227 were classified as Types 1 and 2. We therefore included diatom fragments in the Minor compositions of both Types 1 and 2 as well. The typical refractive index of opal, a component of diatom shells, lies in the range 1.41–1.46^37^. Diatom shells also contain numerous internal cavities (e.g., Fig. 3a). The Im*S*/Re*S* ratio of particles with such shapes tends to be lower than that of spherical (compact) particles^33^. The *S* data points for such particles would lie to the right of the theoretical curve corresponding to *m* = 1.46. This expectation was consistent with the *S* data points of NIES4227, which were distributed in Type 0 and 1 regions to the right of the theoretical curve corresponding to *m* = 1.43. It is unclear why ~ 20% of the data points were distributed in the Type 2 region, which is bounded by *m* = 1.60 and *m* = 1.43, but it is possible that particles other than diatoms in the culture sample were contributing to the distribution in this region.

We considered carbonaceous particles to be a Major composition of Type 1. In the analyses of CAS34 and CAS35 samples, the highest number of particles were classified into Type 1 in the CAS measurements and into carbonaceous in the SEM analyses. Moreover, the peaks of the size-resolved concentrations of the Type 1 particles derived from the CAS measurements closely resembled those of the relative size-resolved concentrations of carbonaceous particles derived from the SEM–EDS analyses (Fig. 3b). In marine environments, SPM is composed primarily of organics^1,38^. Within the size range of particles analyzed in this study (0.2–5.0 µm), the composition of organic particles included colloidal particles, bacteria, cyanobacteria, eukaryotic picoplankton, and detritus^21,39^. Aas^37^ has calculated the refractive index of phytoplankton, assuming a typical volume fraction of water (0.6–0.8), to be 1.377–1.417. The close alignment of this range of refractive indices with the lower limit (the theoretical curve with *m* = 1.38) and upper limit (*m* = 1.43) of Type 1 on the complex *S* plane supports our hypothesis that carbonaceous particles are a Major composition of Type 1. Snomax (bacteria), which do not contain chlorophyll, are expected to have a lower refractive index than phytoplankton, and that lower refractive index would probably lead to a lower Im*S*/Re*S* ratio, which might explain why data points for Snomax were located in the regions of both Types 0 and 1.

We assumed mineral dust to be a Major composition of Type 2 based on the *S* data for the NX illite, quartz, and calcium carbonate samples (Fig. 2e,f). Note that the data points for NX illite were distributed on the boundary between Types 1 and 2. The lower number concentration of particles in Type 2 compared to Type 1 in the SPM samples might have obscured the *S* clusters of mineral dust and led to uncertainty in identification of the composition of Type 2 particles. Although calcium carbonate is a ubiquitous component of mineral dust, it is also a component of coccoliths, which form the shells of coccolithophores. For this reason, we include coccolith as a Possible composition of Type 2. Further measurements of coccolithophore cultures are necessary to confirm the Type associated with coccoliths.

We categorized iron oxides and BC as Major components of Types 3 and 4, respectively, because reference data for iron oxides belonged to Type 3, and data for atmospheric BC smaller than ~ 0.4 µm during the same research cruise^25^ belonged to Type 4. We note that the amounts of SPM categorized as Type 3 or 4 were much lower than those of other Types and that the data points for large BC (> ~ 0.4 µm) occasionally overwhelmed those distributed in the Type 3 region, resulting in a technical difficulty for the characterization of particle compositions for Types 3 and 4 contained in the seawater samples.

### Size distribution and depth profile of SPM measured by CAS

#### Size distribution

**Fig. 4:**
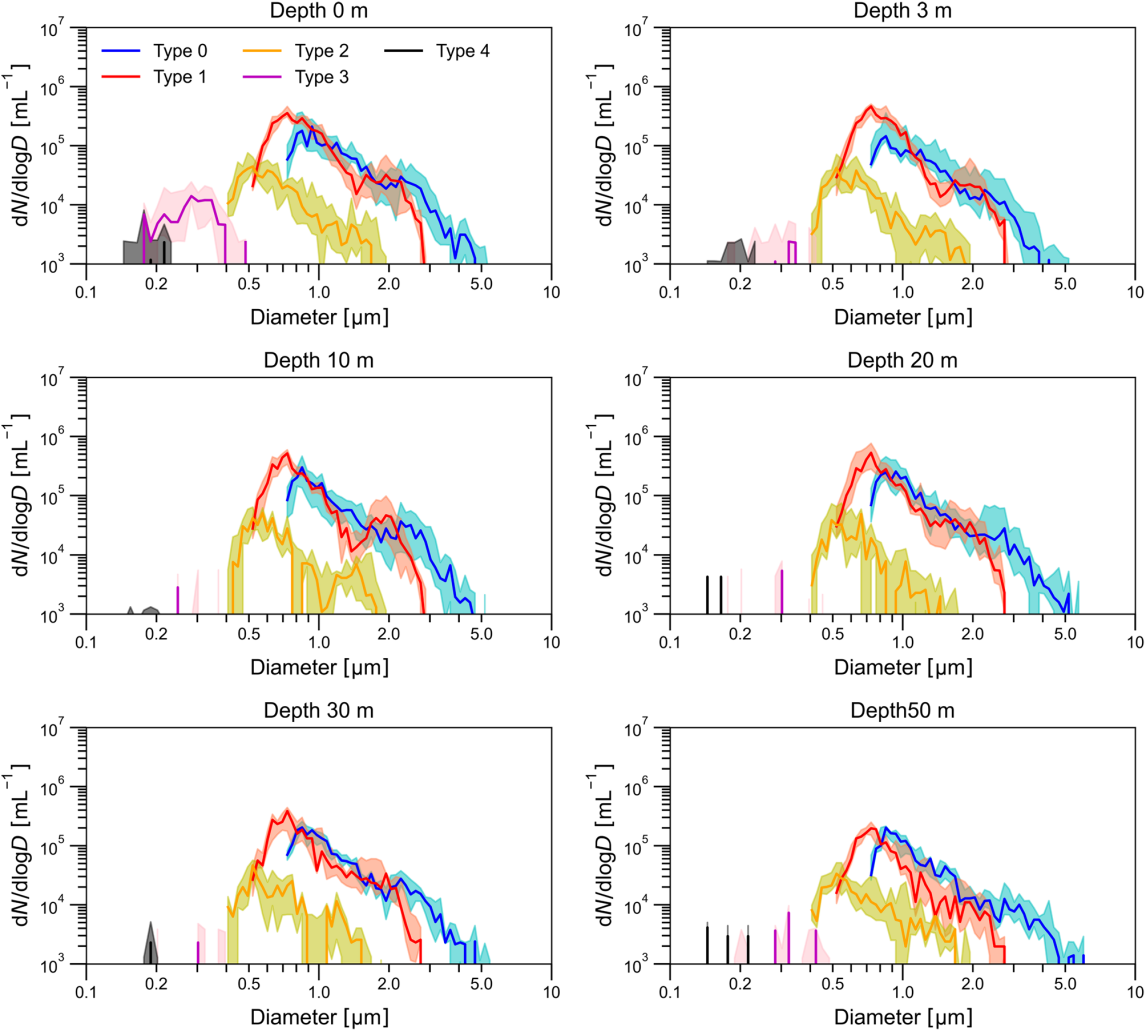
Size-resolved number concentrations of Type 0–4 particles obtained by the in situ measurements shown by depth. Solid line and shaded area represent median and 25–75 percentile values, respectively.

Figure 4 shows size-resolved number concentrations of each type by depth for all in situ measurements. Following the CAS34 and CAS35 analyses, we determined particle sizes for Type 4 as BC and Type 0–3 as non-absorbing spheres. The sizes of Types 0, 1, 2, 3, and 4 were approximately 0.7–5.0, 0.5–3.0, 0.7–2.0, 0.2–0.5, and 0.2 µm, respectively. The peak sizes of Types 0, 1, and 2 were approximately 0.8, 0.7, and 0.5 µm, respectively. Types 0–2 exhibited relatively consistent size-resolved concentration shapes across different depths. Types 3 and 4 showed no distinct peak because of their low concentrations, and they were seldom detected below the surface. Supplementary Fig. 6 shows the size-resolved volume concentrations d*V*/dlog*D* obtained by multiplying d*N*/dlog*D* by π*D*^3^/6. The volume concentrations for Types 3 and 4 were too low to be visible in the figure because the values were more than two orders of magnitude lower than those for Types 0 and 1. For Types 0–2, the peaks observed in the size-resolved number concentrations became less distinct, and significant volume concentrations were even observed near the upper limit of the size-resolved concentrations (~ 5 µm).

We further investigated the difference of size-resolved number concentrations between the filter and in situ measurements for Types 0–2 (Supplementary Fig. 7). The ratio of d*N*/dlog*D* determined by the filter and in situ measurements for Types 0–2 increased and exceeded 1.0 as the particle size approached the lower limits of the CAS particle sizing. This feature was particularly evident in Type 1, where the ratio decreased notably as the particle size increased beyond ~ 1.0 µm. This pattern suggested that the filtration and sonication used for filter measurements might have some impact on the number-size distribution of fragile carbonaceous particles^38,40^. Filter measurements, however, have the advantage of being able to yield concentrated SPM suspensions and more data points than do in-situ measurements (Fig. 2).

#### Comparison of SPM concentrations with turbidity and chlorophyll concentrations

**Fig. 5:**
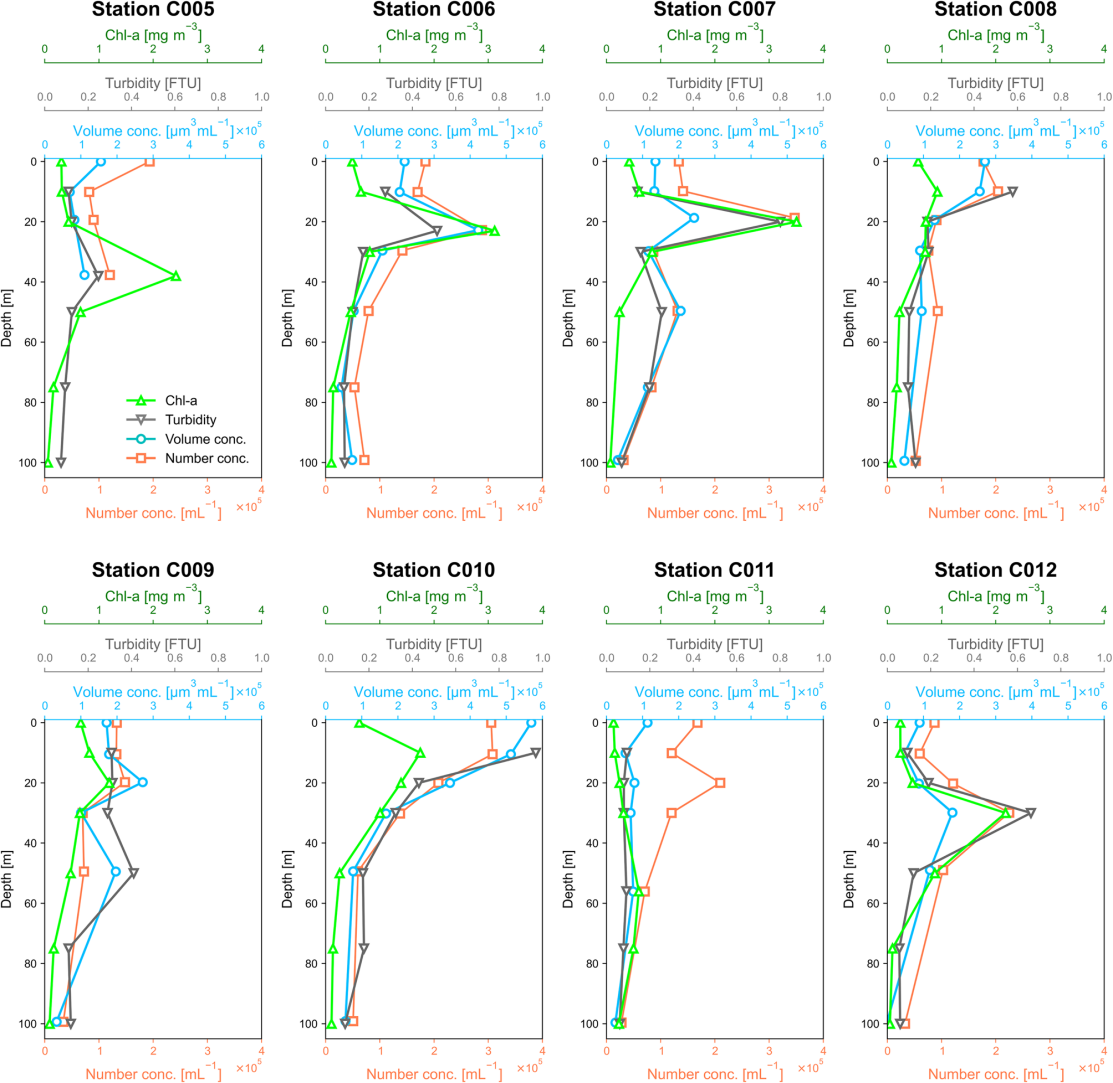
Vertical profiles of number and volume concentrations of SPM obtained by the in situ measurement, comparing with the vertical profiles of turbidity and Chl-*a* concentration.

Figure 5 shows vertical profiles of SPM number and volume concentrations determined by in situ measurements, and vertical profiles of Chl-*a* concentrations and turbidity at eight CTD stations (C005–C012). The volume concentration was derived by integrating the d*V*/dlog*D* over the whole size range obtained by s- and l-channel measurements. The Chl-*a* concentrations were lower at station C011 than at other stations because C011 was located in a warm eddy detached from the Kuroshio Extension. The high Chl-*a* concentration at C005, where the T–S diagram was close to that of Kuroshio Current, might have been due to the intrusion of nutrient-rich Oyashio water. The subsurface chlorophyll maximum layers (SCMLs), which were created mainly by a combination of light attenuation and the vertical gradient of nutrient concentrations, were clearly apparent at depths of 10–60 m. The depths of the SCML were typical for waters ranging from inshore to offshore^41^. The number and volume concentrations of SPM correlated relatively well with turbidities, indicating that CAS-derived volume concentrations were properly quantified. Furthermore, the peak depths of SPM concentrations were located near the depths of the SCML. At C011, there was a clear peak of the SPM number concentration at 20 m, but the peaks of volume concentration and Chl-*a* were weak, and there was no apparent peak of turbidity. More sub-micron particles and fewer super-micron particles were observed at a depth of 20 m at C011 than at the other stations (not shown). We could not confirm this abundance of sub-micron particles by the particle volume dependence of the Chl-*a* concentrations or the particle surface area or volume dependence of the turbidity.

**Fig. 6:**
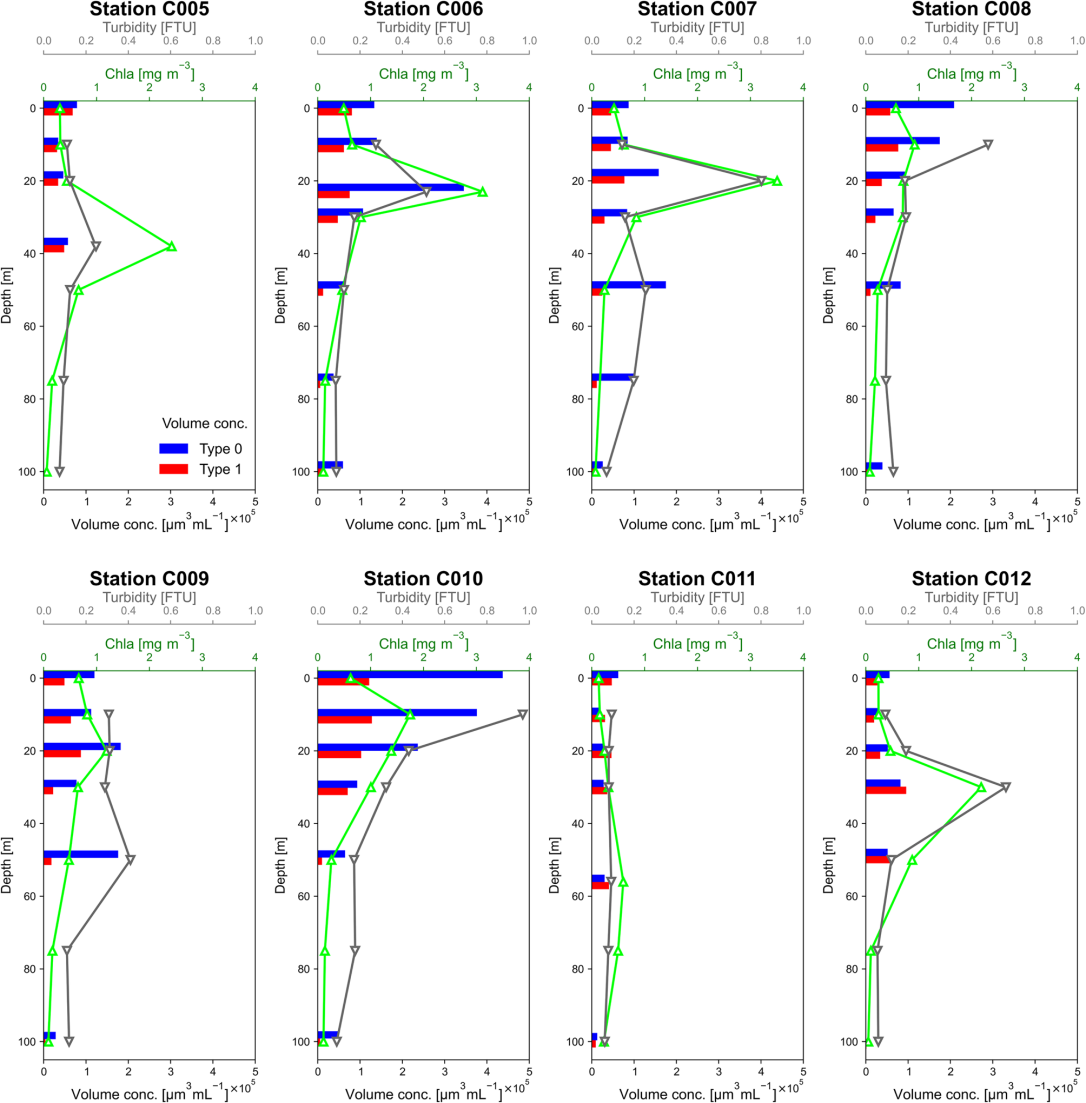
Vertical profiles of volume concentrations for Types 0 and 1 obtained by the in situ measurements, comparing with the vertical profiles of turbidity and Chl-*a* concentration. Types 2–4 are not shown due to their low concentrations.

Figure 6 shows vertical profiles of SPM volume concentrations for Types 0 and 1. Types 2–4 are not shown because their concentrations were low. At depths shallower than the SCML, the distributions of the concentrations of Type 1 were similar to those of the Chl-*a* concentrations, although there were not strong peaks of Type 1 at the depths of the SCMLs in some profiles (e.g., C005, C006, C007, and C010). At greater depths, both the Type 1 and Chl-*a* concentrations decreased sharply. The patterns of the concentrations of Type 0 were similar to those of turbidity. Supplementary Fig. 8 shows the correlations between the Chl-*a* concentrations and the SPM volume concentrations by Type for all samples measured in this study. The *R*^2^ values (*p* values) for the correlations associated with the linear regressions were 0.48 (1.9 × 10^−8^), 0.64 (2.6 × 10^−12^), 0.37 (3.3 × 10^−6^), 0.02 (0.096), and 0.00 (0.35) for Type 0, 1, 2, 3, and 4, respectively. These values suggested that the origins of some of the Type 0–2 particles may have been phytoplankton, whereas Type 3 and 4 particles were unrelated to phytoplankton. This interpretation is consistent with our hypothesis that the compositions of Types 0–2 included carbonaceous and diatom fragments, whereas those of Types 3 and 4 did not (Table 1).

#### Type 3 and Type 4 particles in the sea surface

**Fig. 7:**
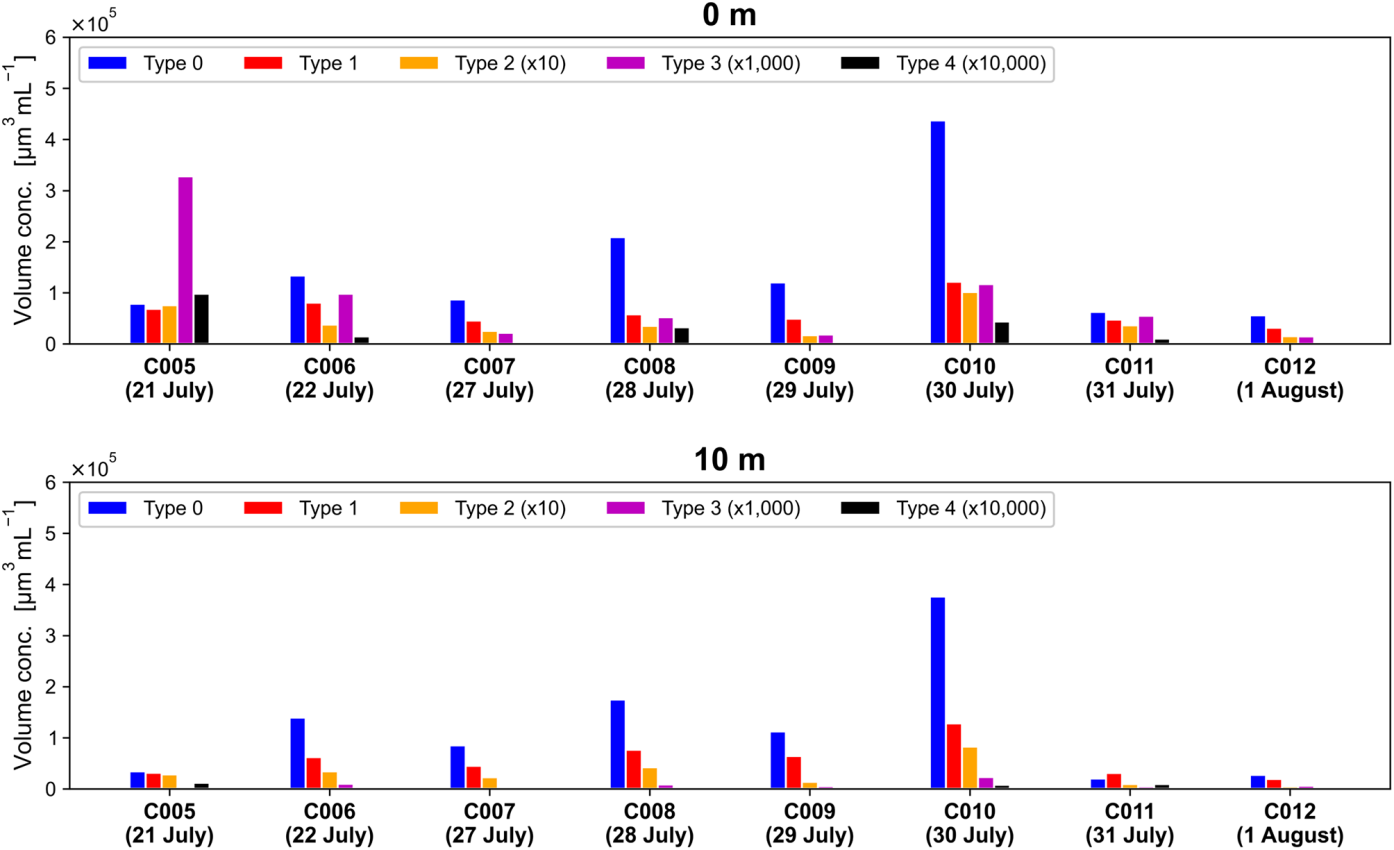
Temporal variations in the volume concentration of each Type of SPM at a depth of 0 m and 10 m. The date are displayed in JST.

Figure 7 shows the volume concentrations of Types 0–4 at a depth of 0 m (sea surface) and a depth of 10 m. The values for Types 2–4 have been magnified to facilitate visual analysis. At all stations, the volume concentrations of Types 3 and 4 were distinctly higher at the sea surface than at a depth of 10 m. The volume concentrations of Types 0 and 1 showed no such tendency. At the sea surface, the highest concentrations of Types 3 and 4 occurred at C005. The Type 3 and 4 particles observed at C005 were likely deposited from the atmosphere via rainfall for the following three reasons. First, iron oxides and BC, the compositions of Types 3 and 4, are known to be water-insoluble components of aerosols^42^. Second, a frontal system associated with precipitation passed through the research area on 20 July, the day before the observations at C005. The resulting precipitation might have formed a freshwater lens at the sea surface^43^, where aerosol particles in the raindrops might have accumulated. Third, the mean (standard deviation) of the 1-min wind speed measured on the vessel from 22:00 on 20 July (JST), after the frontal system had passed, to 10:00 on 21 July (JST), when observations were made at C005, was 2.1 (0.95) m s^−1^. Such weak winds would have probably not perturbed the near-surface stratification^44^. During the observations at stations C007 to C012, more than three days had passed since the passage of the last frontal system (23 July). However, we nevertheless observed fluctuations of the concentrations of Type 3 and 4 particles at the sea surface. It is possible that Type 3 and 4 particles remained in the surface layer such as microlayer^45^ around these stations.

Mineral dust is a major component of water-insoluble aerosols, and it is possible that it was deposited on the sea surface through precipitation. The volume concentration of Type 2 particles was higher at station C005 than at C006, similar to the volume concentrations of Types 3 and 4 (Fig. 7). This similarity suggests that some Type 2 particles, likely mineral dust, might also have been deposited from the atmosphere. However, unlike Types 3 and 4, Type 2 particles were not concentrated at the ocean surface. The correlation of Type 2 particle concentration with Chl-*a* concentration suggests that particles of biological origin, such as carbonaceous particles and coccolithophores, may also be classified as Type 2.

## Conclusions

In this study, we evaluated the capability of CAS to characterize the SPM in seawater. We confirmed that the size-resolved concentrations of SPM derived from the CAS measurements were reasonably consistent with the results of Coulter Counter measurements. The number and volume concentrations of SPM derived by CAS were also well correlated with turbidity. These results indicated that CAS could provide reliable quantification of SPM in the ocean. We further classified the CAS-derived SPM into five major types (Type 0 to Type 4). Based on the comparison with the reference sample’s complex amplitude data and SEM–EDS analysis, we propose that these Types could be attributed mainly to diatom fragments, carbonaceous materials (likely organic matter), mineral dusts, iron oxides, or black carbon.

The majority of SPM in the ocean is thought to consists of organic particles^1,38,46,47^. Our results indicate that particles in Types 0 and 1, which were likely to be dominated by carbonaceous particles and diatom fragments, were prevalent in seawater. The volume concentrations of Type 0 and 1 particles correlated relatively well with the turbidity and Chl-*a* concentration, which is consistent with our hypothesis that the main compositions of these Types include carbonaceous (likely organic matter). Type 2–4 particles were less abundant compared to Type 0 and 1 particles. Nevertheless, CAS could observe relatively high amounts of Type 2–4 particles at sea surface, which were probably deposited from the atmosphere. Although there are still some technical difficulties with the boundaries for classifying particle Types in the complex *S* plain and there could be uncertainties in identifying particle compositions, these results suggested that CAS measurements have potential to quantify size-resolved concentrations of SPM from both biogenic sources (carbonaceous and diatom fragments) and external sources (mineral dust, iron oxides, and black carbon) in the size range of 0.2–5.0 µm in diameter. The classification of these compositions is important for ocean biogeochemical studies because each composition has distinctive features. For example, organic particles play important role in carbon cycle^2^, diatoms affect silicon and carbon cycles through biological pumps^48^, mineral dust and iron oxides have potential to supply micronutrient to phytoplankton^49,50^, and BC can adsorb organic particles and alert their size^45^. Further development of CAS measurements is expected to be useful for observing the abundance and compositions of SPMs in the ocean. In addition, further measurements of SPM in various sea areas worldwide are needed to assess whether the CAS measurements are universally applicable.

### Supplementary Information

Supplementary Figures.

## Data Availability

The size-resolved concentrations of SPM used in this study are available at https://www.jamstec.go.jp/datadoi/doi/10.17596/0003381.html. Raw data of individual particles are available from the corresponding author.
