# Supplementary material for: Analysis of oceanic suspended particulate matter in the western North Pacific using the complex amplitude sensor
Source: Sci Rep. 2024 Aug 29;14:20055. doi: 10.1038/s41598-024-70683-1 (PMC11362335; doi:10.1038/s41598-024-70683-1)
Supplement: Supplementary file 1 — Supplementary Figures. [file 41598_2024_70683_MOESM1_ESM.pdf]

Supplementary Information

Analysis of oceanic suspended particulate matter in the western North Pacific using the complex amplitude sensor

Atsushi Yoshida[1, *], Yutaka Tobo[1,2], Kouji Adachi[3], Nobuhiro Moteki[4], Yoshimi Kawai[5], Kosei Sasaoka[5] & Makoto Koike[6]

[1]National Institute of Polar Research, Tachikawa, Tokyo, Japan. [2]Graduate Institute for Advanced Studies, SOKENDAI, Tachikawa, Tokyo, Japan. [3]Meteorological Research Institute, Tsukuba, Ibaraki, Japan. [4]Tokyo Metropolitan University, Hachioji, Tokyo, Japan. [5]Japan Agency for Marine-Earth Science and Technology, Yokosuka, Kanagawa, Japan. [6]The University of Tokyo, Tokyo, Japan.

Correspondence to Atsushi Yoshida (email: yoshida.atsushi@nipr.ac.jp)

This file includes:

Supplementary Figures 1–8.

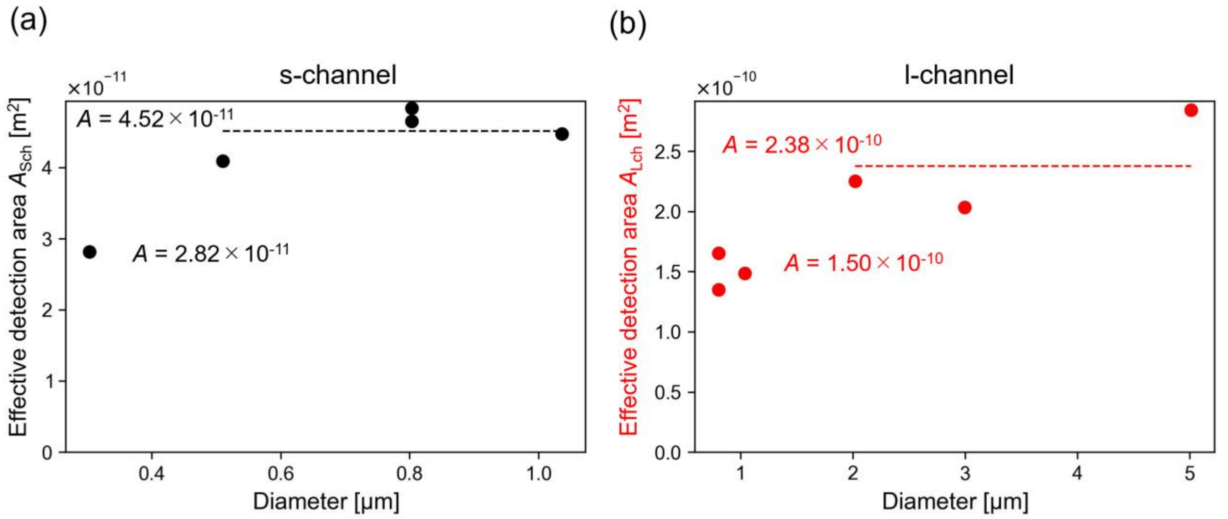

Supplementary Figure 1. Effective detection area A determined by laboratory experiments using PSL with various size. Dashed lines denote mean values for PSL larger than 0.4 and 1.5 μm for s- and l-channels, respectively.

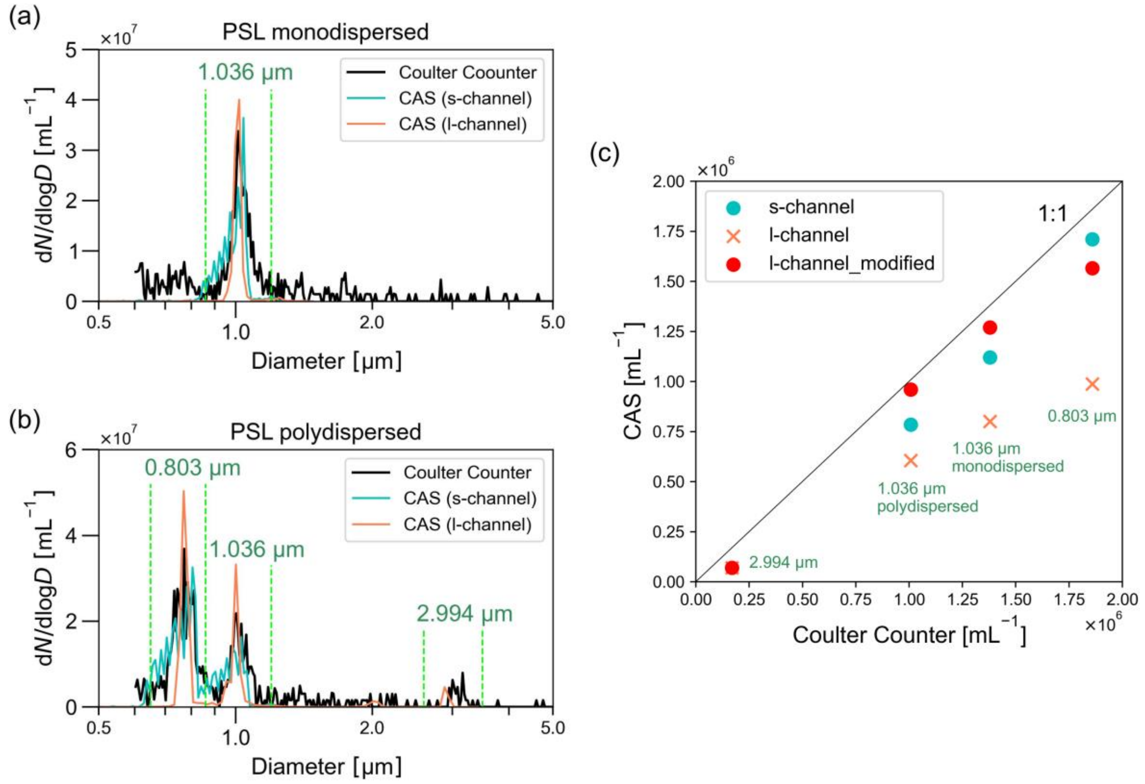

Supplementary Figure 2. Size-resolved concentrations of (**a**) monodispersed and (**b**) polydispersed PSL suspensions measured by CAS and Coulter Counter. Vertical green lines describe thresholds for separating PSL with sizes of D = 0.803, 1.036, and 2.994 μm. (**c**) Correlation of number concentration of PSL in each size range measured by CAS and Coulter Counter.

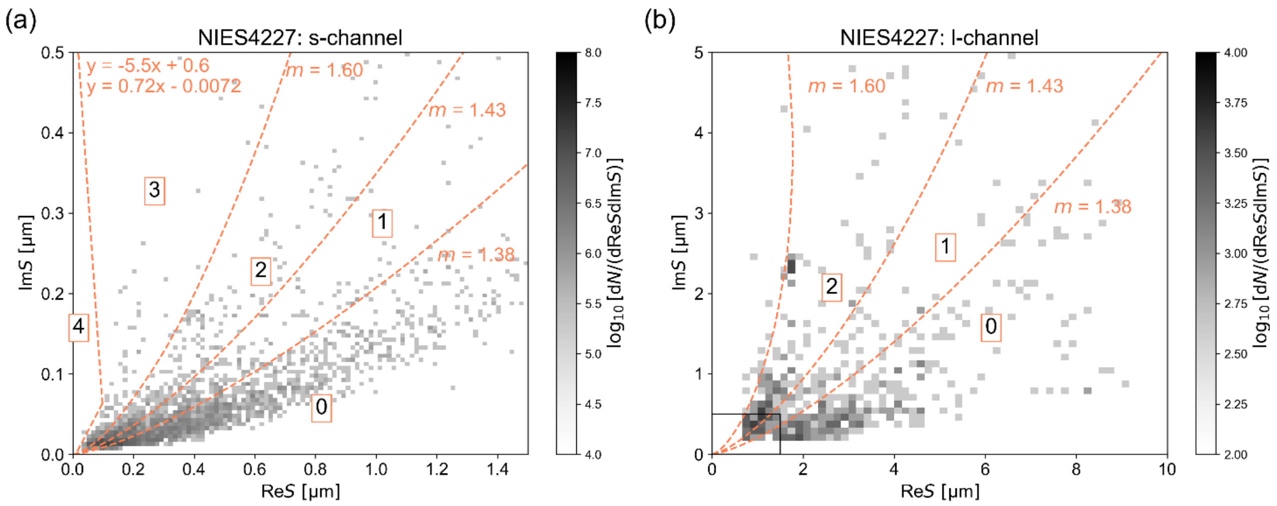

Supplementary Figure 3. Distribution of *S* data points of NIES4227.

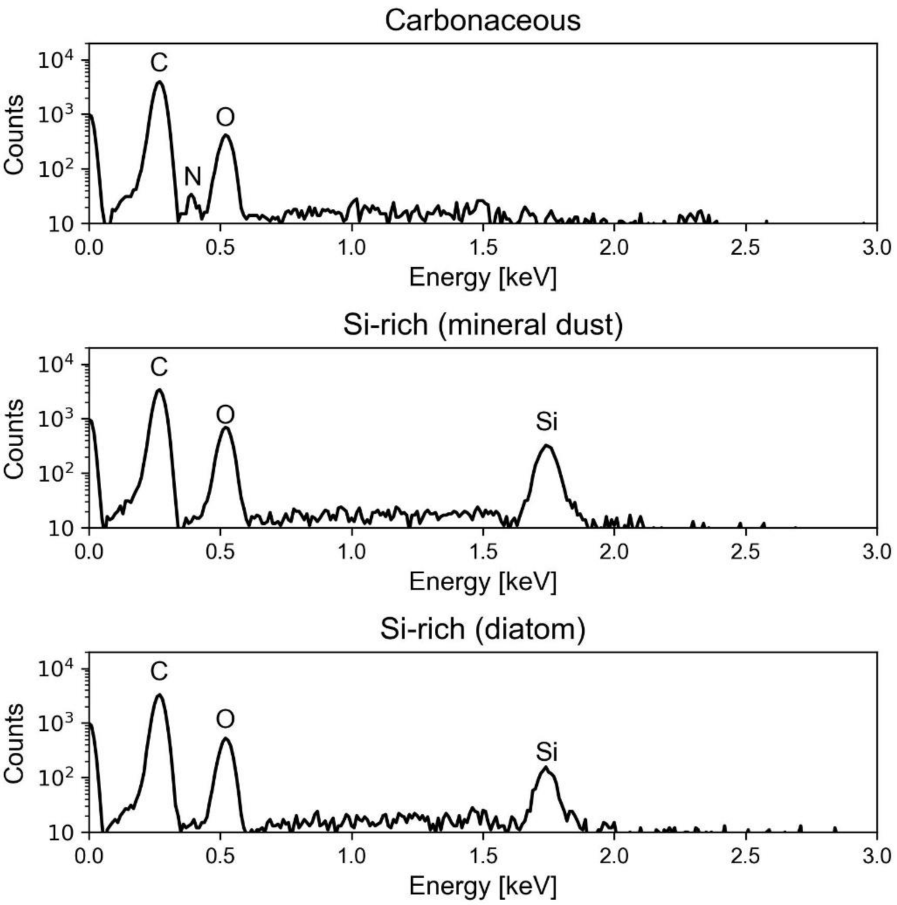

Supplementary Figure 4. Representative SEM-EDS spectra for CAS35.

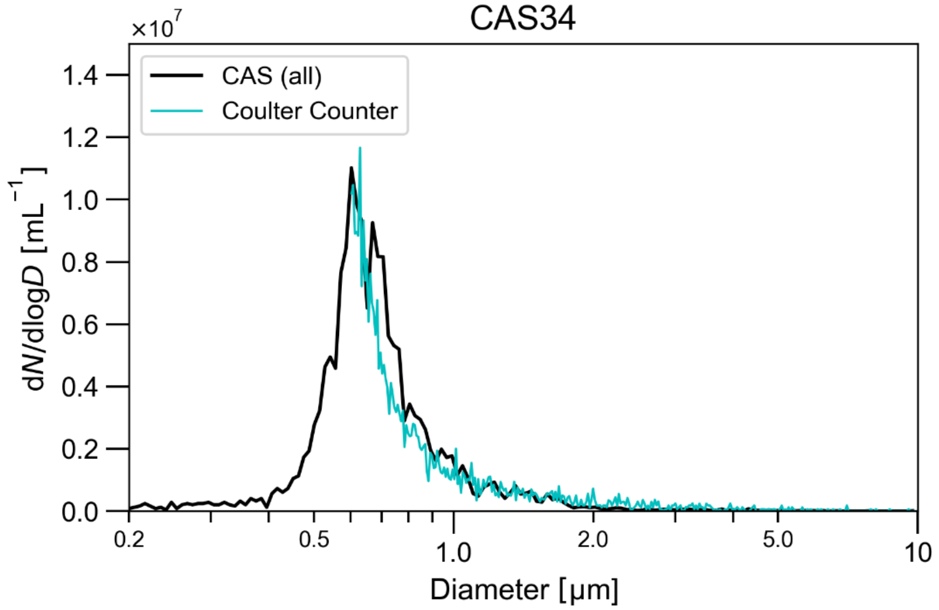

Supplementary Figure 5. Comparison of size-resolved number concentrations of CAS34 sample determined by CAS and Coulter Counter.

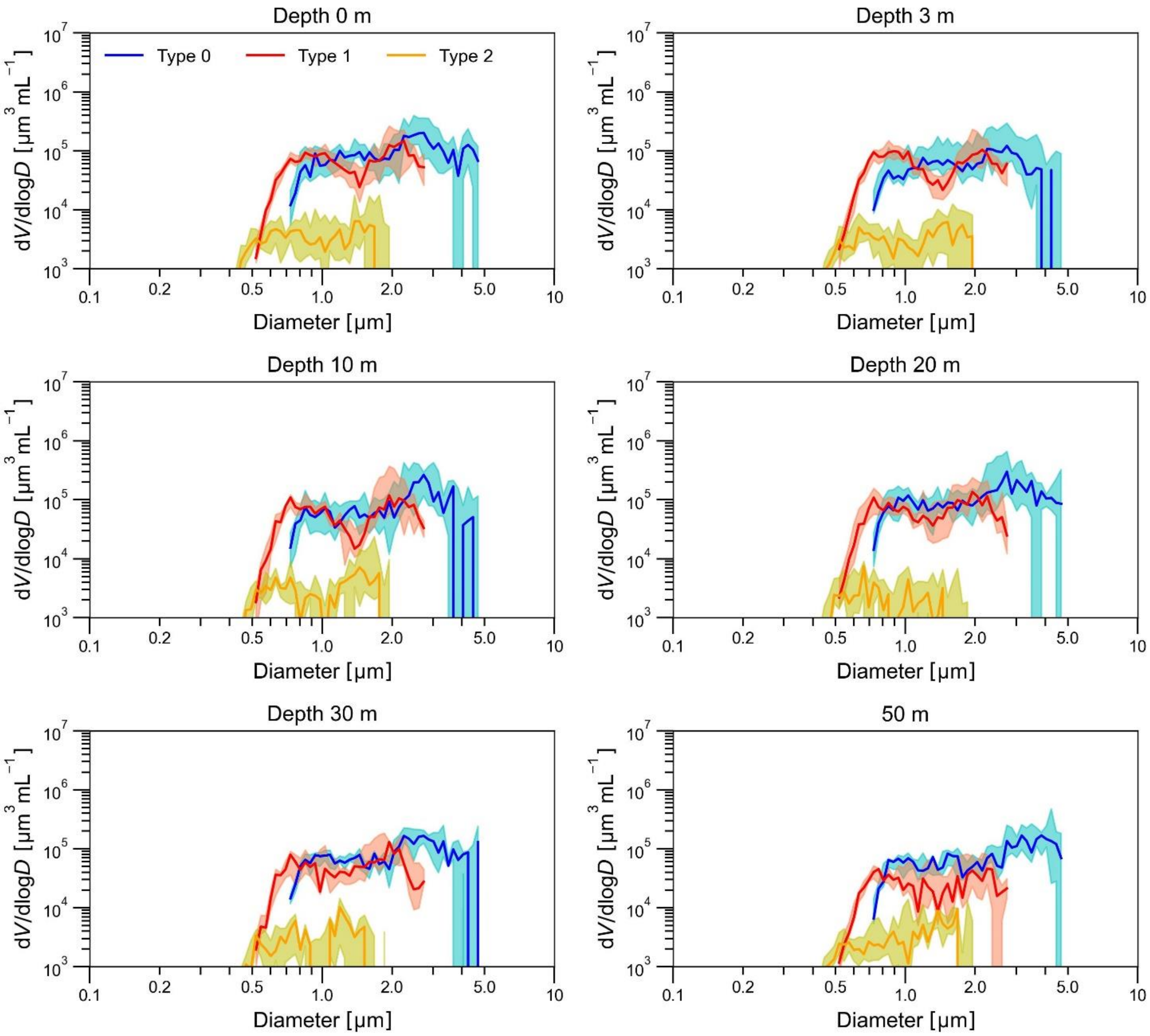

Supplementary Figure 6. Size-resolved volume concentrations of Type 0–2 particles obtained by the in situ measurements shown by depth. Solid line and shaded area represent median and 25–75 percentile values, respectively.

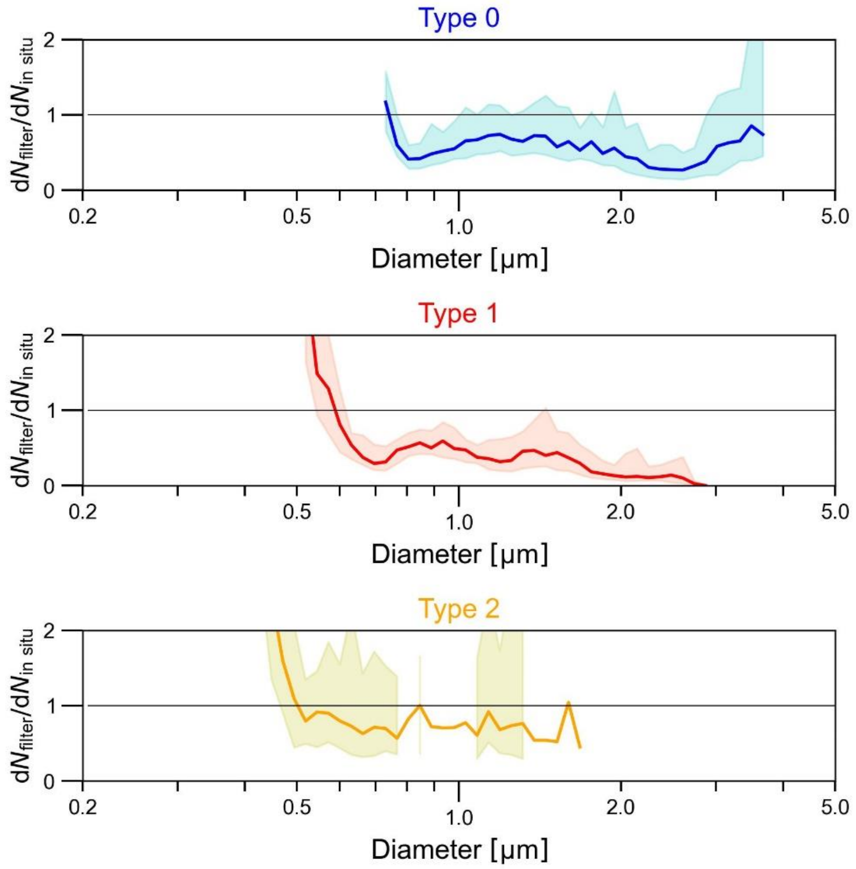

Supplementary Figure 7. Ratios of d*N*/dlog*D* for SPM sample measured by the filter and in situ methods. Solid line and shaded area represent median and 25–75 percentile values, respectively.

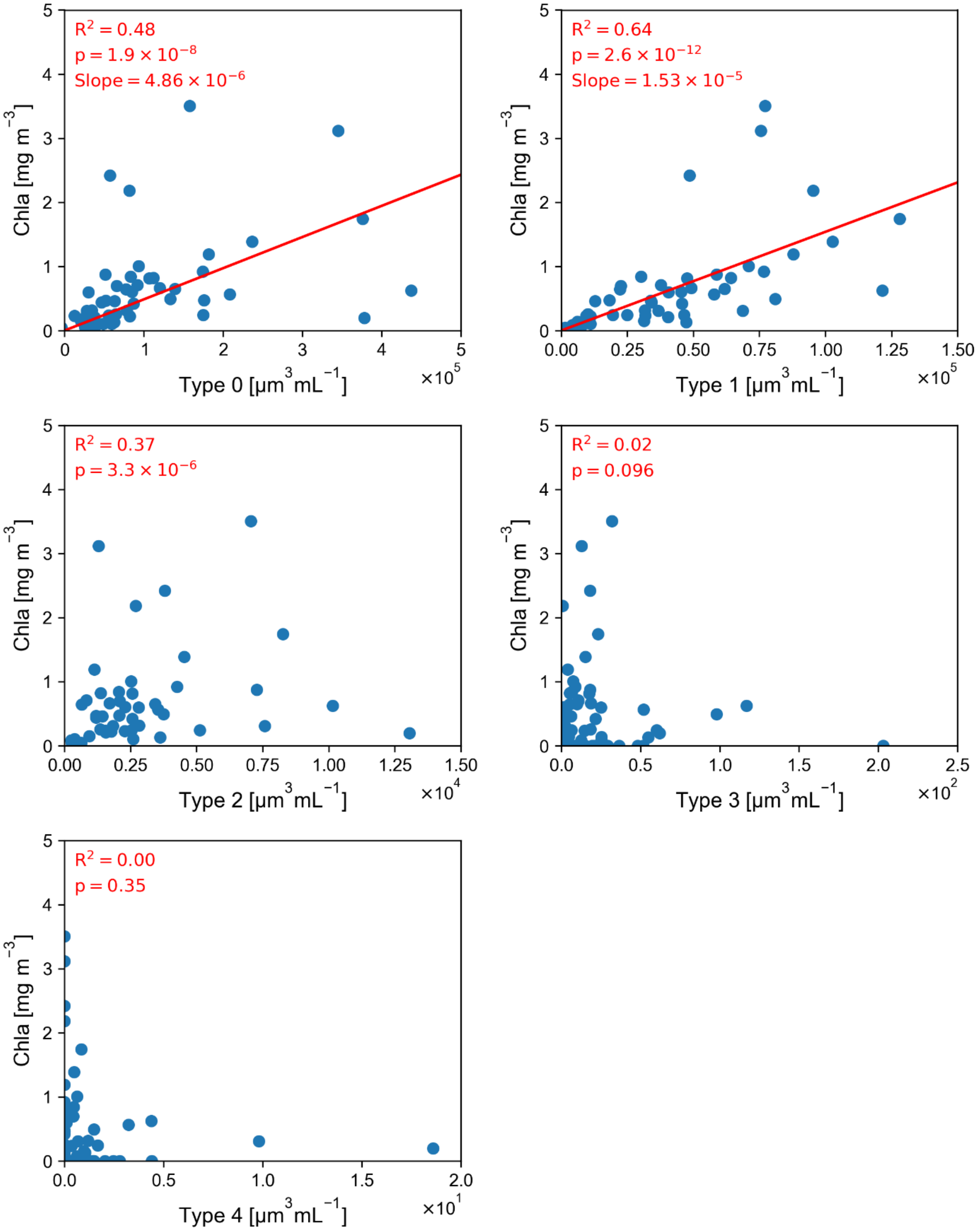

Supplementary Figure 8. Scatterplot of the Chl-a concentration and volume concentration SPM by each type. The R^2 values and slopes (only for Type 0 and 1) determined by linear regression were also denoted.
